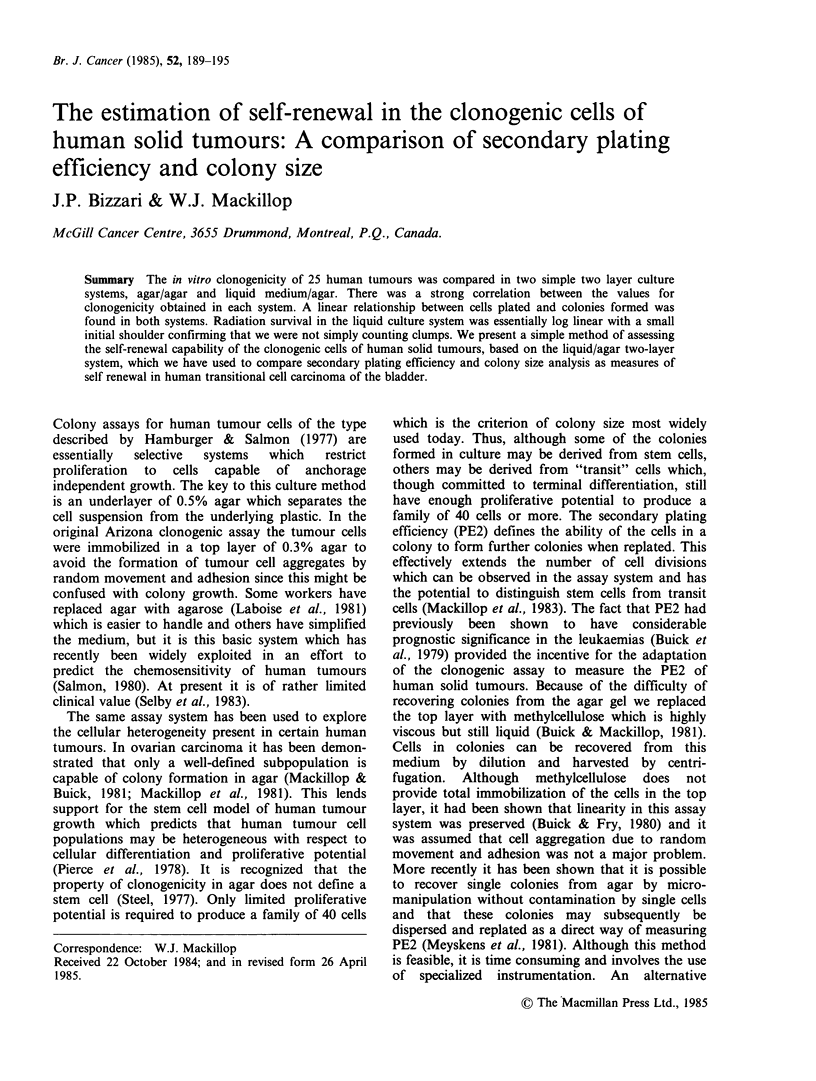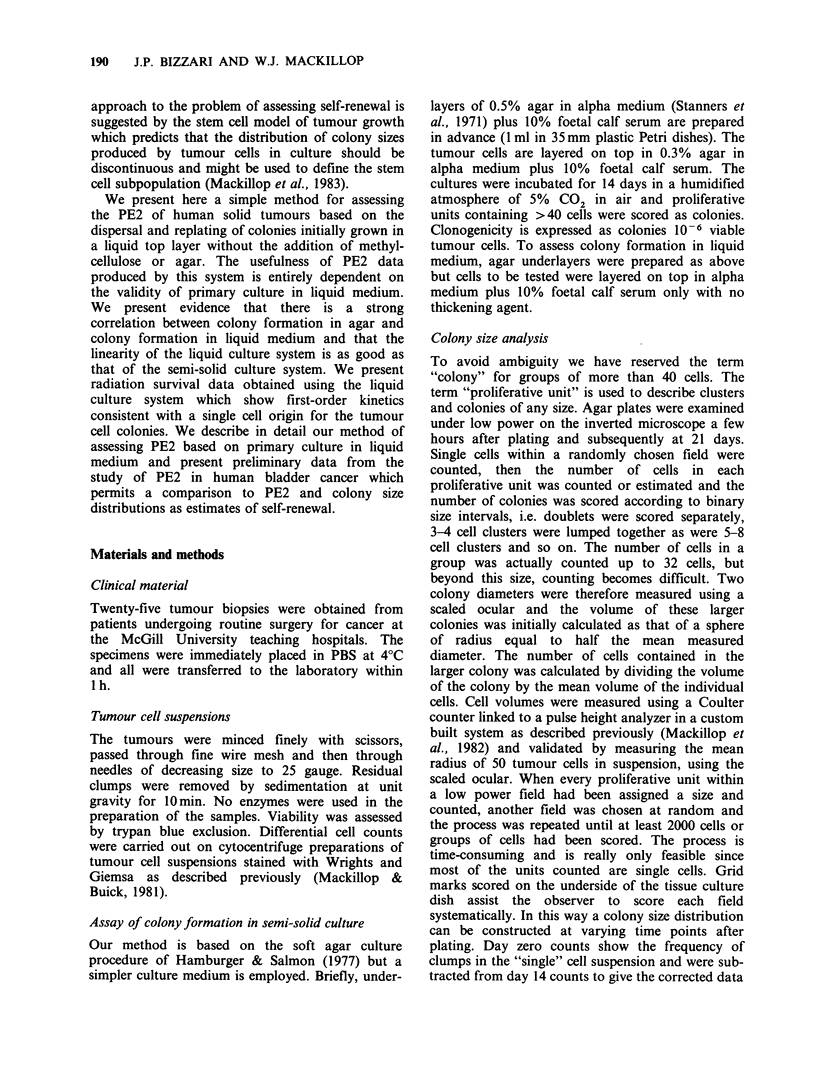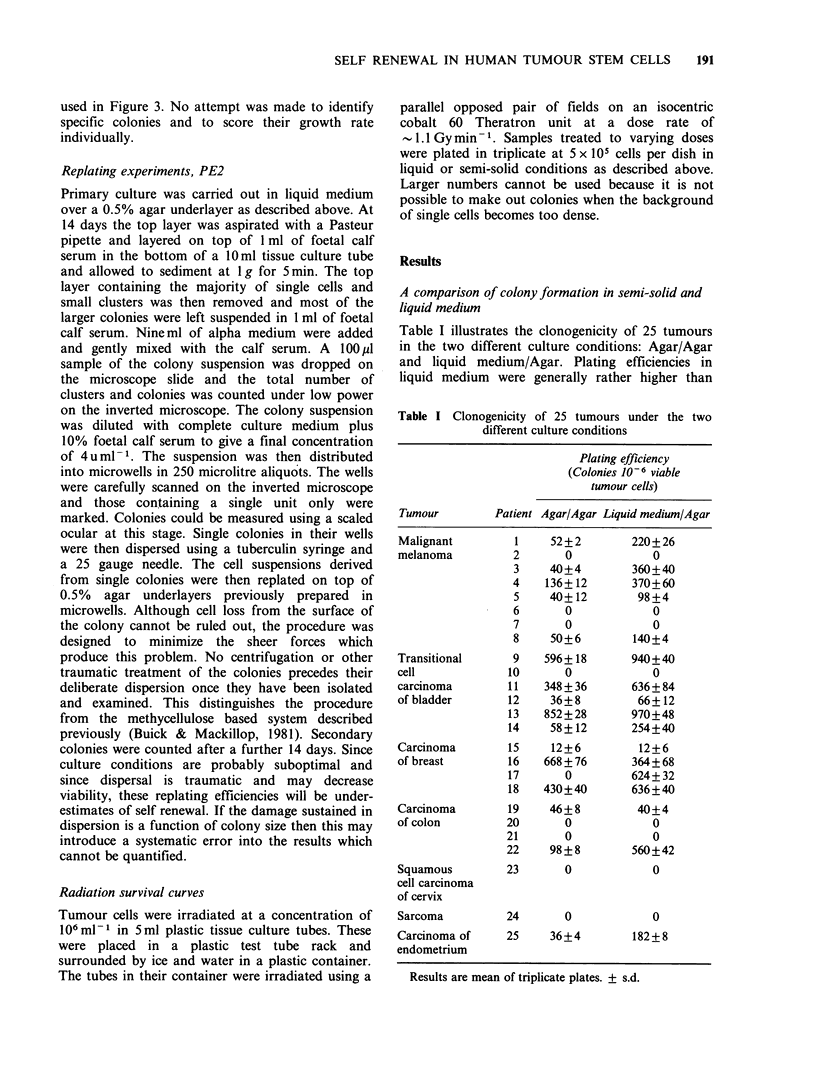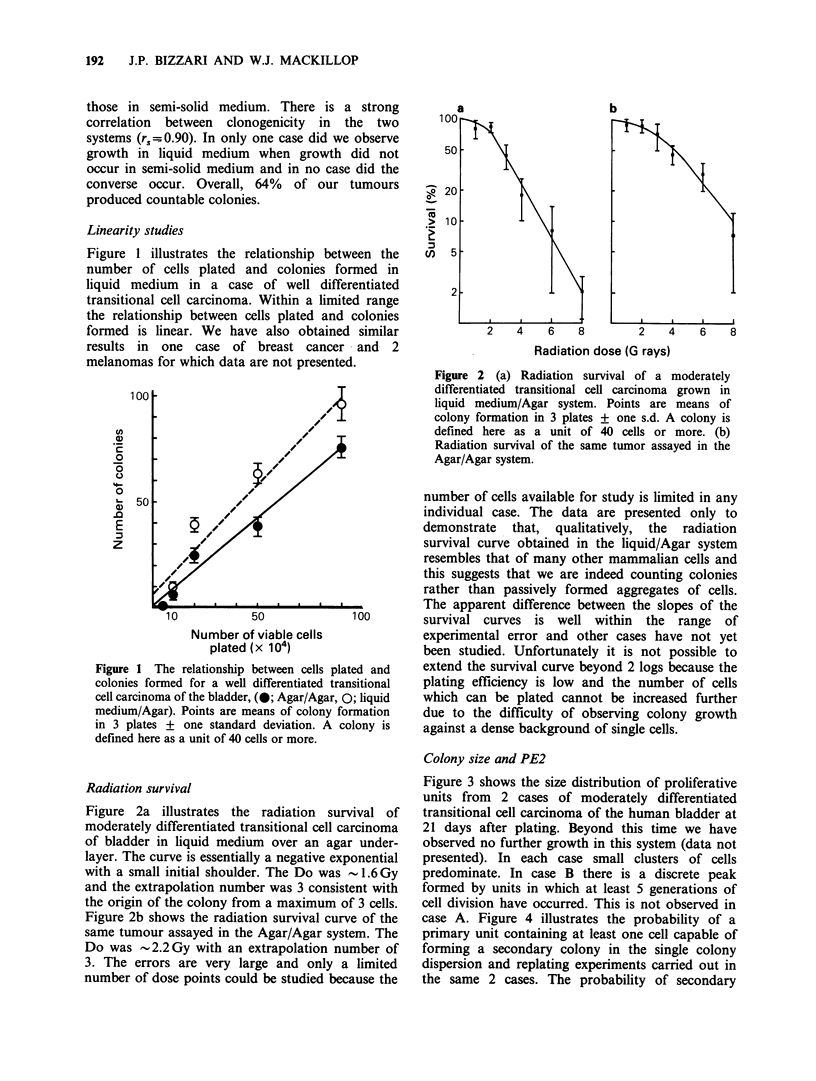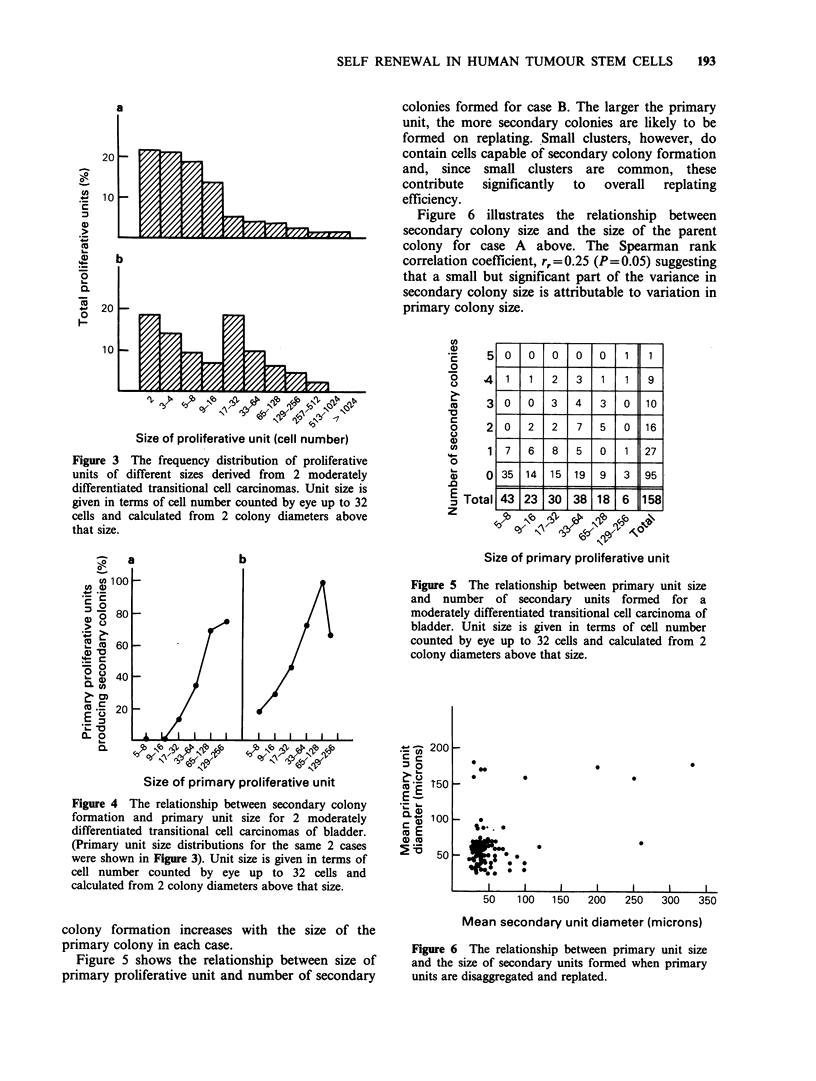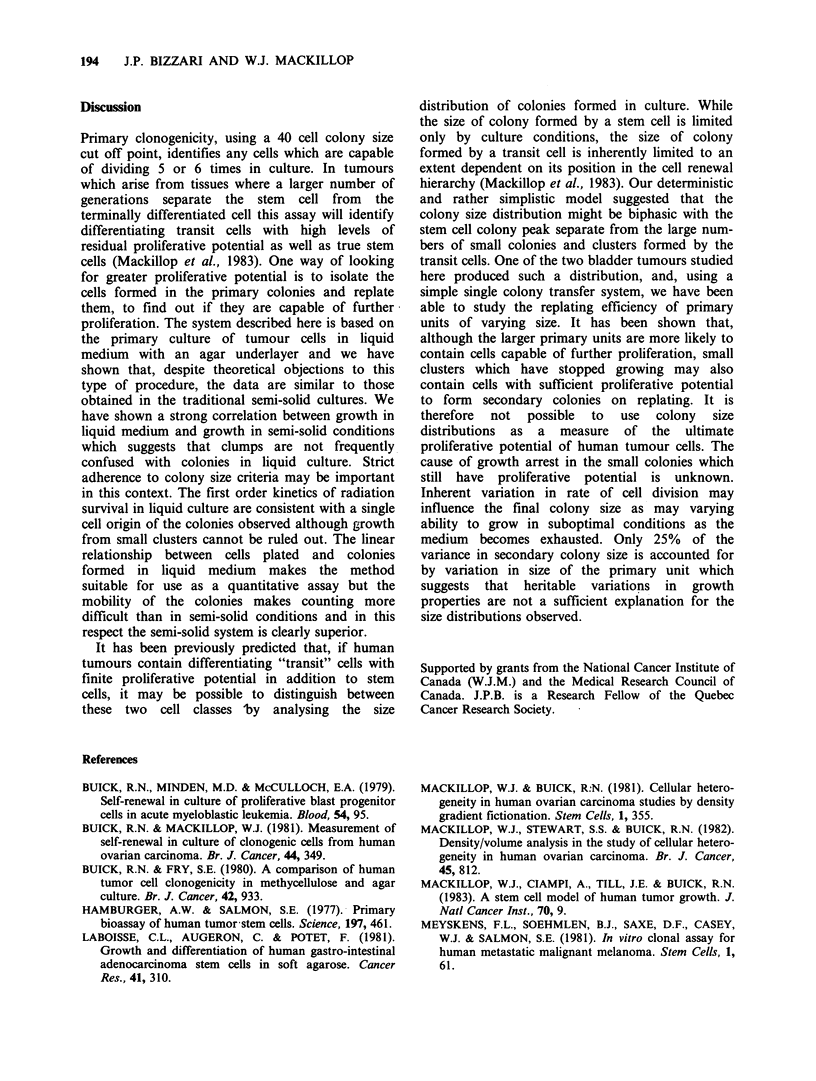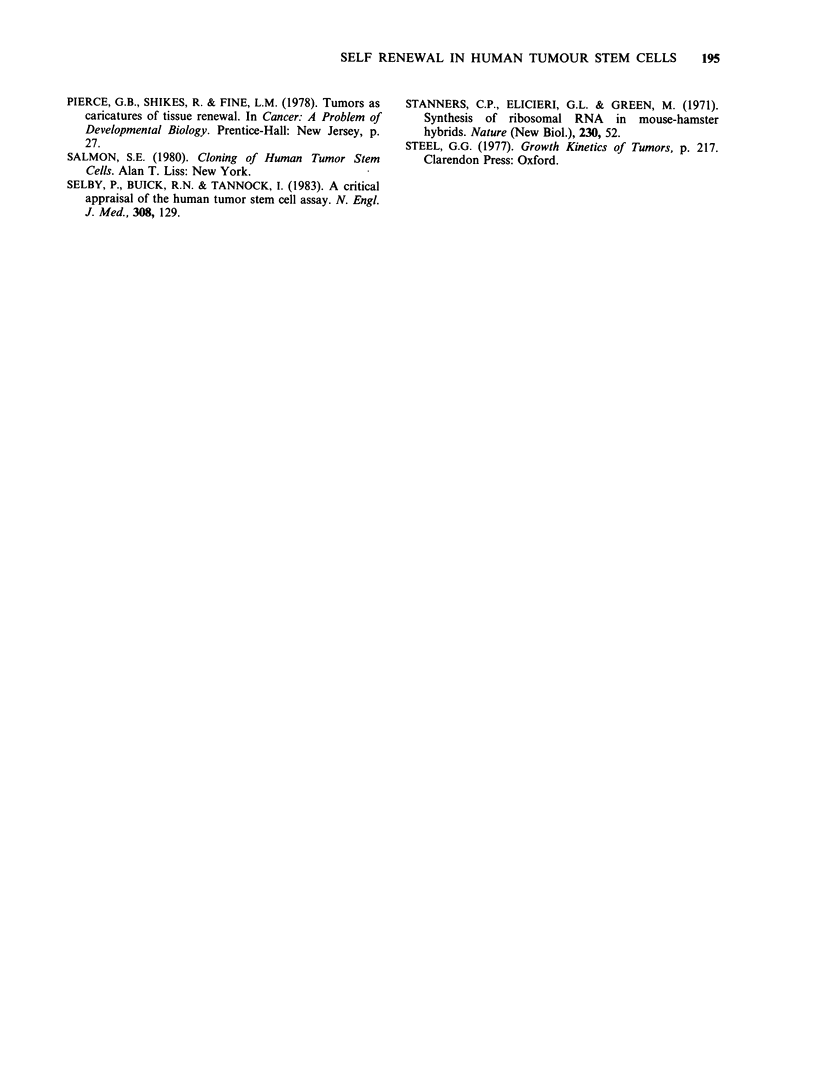# The estimation of self-renewal in the clonogenic cells of human solid tumours: a comparison of secondary plating efficiency and colony size.

**DOI:** 10.1038/bjc.1985.177

**Published:** 1985-08

**Authors:** J. P. Bizzari, W. J. Mackillop

## Abstract

The in vitro clonogenicity of 25 human tumours was compared in two simple two layer culture systems, agar/agar and liquid medium/agar. There was a strong correlation between the values for clonogenicity obtained in each system. A linear relationship between cells plated and colonies formed was found in both systems. Radiation survival in the liquid culture system was essentially log linear with a small initial shoulder confirming that we were not simply counting clumps. We present a simple method of assessing the self-renewal capability of the clonogenic cells of human solid tumours, based on the liquid/agar two-layer system, which we have used to compare secondary plating efficiency and colony size analysis as measures of self renewal in human transitional cell carcinoma of the bladder.


*Br. J. Cancer* (1985), **52**, 189–195

### The estimation of self-renewal in the clonogenic cells of human solid tumours: A comparison of secondary plating efficiency and colony size

J.P. Bizzari & W.J. Mackillop

*McGill Cancer Centre, 3655 Drummond, Montreal, P.Q., Canada.*

**Summary** The *in vitro* clonogenicity of 25 human tumours was compared in two simple two layer culture systems, agar/agar and liquid medium/agar. There was a strong correlation between the values for clonogenicity obtained in each system. A linear relationship between cells plated and colonies formed was found in both systems. Radiation survival in the liquid culture system was essentially log linear with a small initial shoulder confirming that we were not simply counting clumps. We present a simple method of assessing the self-renewal capability of the clonogenic cells of human solid tumours, based on the liquid/agar two-layer system, which we have used to compare secondary plating efficiency and colony size analysis as measures of self renewal in human transitional cell carcinoma of the bladder.

Colony assays for human tumour cells of the type described by Hamburger & Salmon (1977) are essentially selective systems which restrict proliferation to cells capable of anchorage independent growth. The key to this culture method is an underlayer of 0.5% agar which separates the cell suspension from the underlying plastic. In the original Arizona clonogenic assay the tumour cells were immobilized in a top layer of 0.3% agar to avoid the formation of tumour cell aggregates by random movement and adhesion since this might be confused with colony growth. Some workers have replaced agar with agarose (Laboise *et al.*, 1981) which is easier to handle and others have simplified the medium, but it is this basic system which has recently been widely exploited in an effort to predict the chemosensitivity of human tumours (Salmon, 1980). At present it is of rather limited clinical value (Selby *et al.*, 1983).

The same assay system has been used to explore the cellular heterogeneity present in certain human tumours. In ovarian carcinoma it has been demonstrated that only a well-defined subpopulation is capable of colony formation in agar (Mackillop & Buick, 1981; Mackillop *et al.*, 1981). This lends support for the stem cell model of human tumour growth which predicts that human tumour cell populations may be heterogeneous with respect to cellular differentiation and proliferative potential (Pierce *et al.*, 1978). It is recognized that the property of clonogenicity in agar does not define a stem cell (Steel, 1977). Only limited proliferative potential is required to produce a family of 40 cells which is the criterion of colony size most widely used today. Thus, although some of the colonies formed in culture may be derived from stem cells, others may be derived from "transit" cells which, though committed to terminal differentiation, still have enough proliferative potential to produce a family of 40 cells or more. The secondary plating efficiency (PE2) defines the ability of the cells in a colony to form further colonies when replated. This effectively extends the number of cell divisions which can be observed in the assay system and has the potential to distinguish stem cells from transit cells (Mackillop *et al.*, 1983). The fact that PE2 had previously been shown to have considerable prognostic significance in the leukaemias (Buick *et al.*, 1979) provided the incentive for the adaptation of the clonogenic assay to measure the PE2 of human solid tumours. Because of the difficulty of recovering colonies from the agar gel we replaced the top layer with methylcellulose which is highly viscous but still liquid (Buick & Mackillop, 1981). Cells in colonies can be recovered from this medium by dilution and harvested by centrifugation. Although methylcellulose does not provide total immobilization of the cells in the top layer, it had been shown that linearity in this assay system was preserved (Buick & Fry, 1980) and it was assumed that cell aggregation due to random movement and adhesion was not a major problem. More recently it has been shown that it is possible to recover single colonies from agar by micromanipulation without contamination by single cells and that these colonies may subsequently be dispersed and replated as a direct way of measuring PE2 (Meyskens *et al.*, 1981). Although this method is feasible, it is time consuming and involves the use of specialized instrumentation. An alternative

Correspondence: W.J. Mackillop
Received 22 October 1984; and in revised form 26 April 1985.

© The Macmillan Press Ltd., 1985

approach to the problem of assessing self-renewal is suggested by the stem cell model of tumour growth which predicts that the distribution of colony sizes produced by tumour cells in culture should be discontinuous and might be used to define the stem cell subpopulation (Mackillop *et al.*, 1983).

We present here a simple method for assessing the PE2 of human solid tumours based on the dispersal and replating of colonies initially grown in a liquid top layer without the addition of methylcellulose or agar. The usefulness of PE2 data produced by this system is entirely dependent on the validity of primary culture in liquid medium. We present evidence that there is a strong correlation between colony formation in agar and colony formation in liquid medium and that the linearity of the liquid culture system is as good as that of the semi-solid culture system. We present radiation survival data obtained using the liquid culture system which show first-order kinetics consistent with a single cell origin for the tumour cell colonies. We describe in detail our method of assessing PE2 based on primary culture in liquid medium and present preliminary data from the study of PE2 in human bladder cancer which permits a comparison to PE2 and colony size distributions as estimates of self-renewal.

### Materials and methods

### *Clinical material*

Twenty-five tumour biopsies were obtained from patients undergoing routine surgery for cancer at the McGill University teaching hospitals. The specimens were immediately placed in PBS at 4°C and all were transferred to the laboratory within 1 h.

### *Tumour cell suspensions*

The tumours were minced finely with scissors, passed through fine wire mesh and then through needles of decreasing size to 25 gauge. Residual clumps were removed by sedimentation at unit gravity for 10 min. No enzymes were used in the preparation of the samples. Viability was assessed by trypan blue exclusion. Differential cell counts were carried out on cytocentrifuge preparations of tumour cell suspensions stained with Wrights and Giemsa as described previously (Mackillop & Buick, 1981).

### *Assay of colony formation in semi-solid culture*

Our method is based on the soft agar culture procedure of Hamburger & Salmon (1977) but a simpler culture medium is employed. Briefly, underlayers of 0.5% agar in alpha medium (Stanners *et al.*, 1971) plus 10% foetal calf serum are prepared in advance (1 ml in 35 mm plastic Petri dishes). The tumour cells are layered on top in 0.3% agar in alpha medium plus 10% foetal calf serum. The cultures were incubated for 14 days in a humidified atmosphere of 5% $CO_2$ in air and proliferative units containing >40 cells were scored as colonies. Clonogenicity is expressed as colonies $10^{-6}$ viable tumour cells. To assess colony formation in liquid medium, agar underlayers were prepared as above but cells to be tested were layered on top in alpha medium plus 10% foetal calf serum only with no thickening agent.

### *Colony size analysis*

To avoid ambiguity we have reserved the term "colony" for groups of more than 40 cells. The term "proliferative unit" is used to describe clusters and colonies of any size. Agar plates were examined under low power on the inverted microscope a few hours after plating and subsequently at 21 days. Single cells within a randomly chosen field were counted, then the number of cells in each proliferative unit was counted or estimated and the number of colonies was scored according to binary size intervals, i.e. doublets were scored separately, 3–4 cell clusters were lumped together as were 5–8 cell clusters and so on. The number of cells in a group was actually counted up to 32 cells, but beyond this size, counting becomes difficult. Two colony diameters were therefore measured using a scaled ocular and the volume of these larger colonies was initially calculated as that of a sphere of radius equal to half the mean measured diameter. The number of cells contained in the larger colony was calculated by dividing the volume of the colony by the mean volume of the individual cells. Cell volumes were measured using a Coulter counter linked to a pulse height analyzer in a custom built system as described previously (Mackillop *et al.*, 1982) and validated by measuring the mean radius of 50 tumour cells in suspension, using the scaled ocular. When every proliferative unit within a low power field had been assigned a size and counted, another field was chosen at random and the process was repeated until at least 2000 cells or groups of cells had been scored. The process is time-consuming and is really only feasible since most of the units counted are single cells. Grid marks scored on the underside of the tissue culture dish assist the observer to score each field systematically. In this way a colony size distribution can be constructed at varying time points after plating. Day zero counts show the frequency of clumps in the "single" cell suspension and were subtracted from day 14 counts to give the corrected data used in Figure 3. No attempt was made to identify specific colonies and to score their growth rate individually.

*Replating experiments, PE2*

Primary culture was carried out in liquid medium over a 0.5% agar underlayer as described above. At 14 days the top layer was aspirated with a Pasteur pipette and layered on top of 1 ml of foetal calf serum in the bottom of a 10 ml tissue culture tube and allowed to sediment at 1 *g* for 5 min. The top layer containing the majority of single cells and small clusters was then removed and most of the larger colonies were left suspended in 1 ml of foetal calf serum. Nine ml of alpha medium were added and gently mixed with the calf serum. A 100 $\mu$l sample of the colony suspension was dropped on the microscope slide and the total number of clusters and colonies was counted under low power on the inverted microscope. The colony suspension was diluted with complete culture medium plus 10% foetal calf serum to give a final concentration of 4 u $ml^{-1}$. The suspension was then distributed into microwells in 250 microlitre aliquots. The wells were carefully scanned on the inverted microscope and those containing a single unit only were marked. Colonies could be measured using a scaled ocular at this stage. Single colonies in their wells were then dispersed using a tuberculin syringe and a 25 gauge needle. The cell suspensions derived from single colonies were then replated on top of 0.5% agar underlayers previously prepared in microwells. Although cell loss from the surface of the colony cannot be ruled out, the procedure was designed to minimize the sheer forces which produce this problem. No centrifugation or other traumatic treatment of the colonies precedes their deliberate dispersion once they have been isolated and examined. This distinguishes the procedure from the methycellulose based system described previously (Buick & Mackillop, 1981). Secondary colonies were counted after a further 14 days. Since culture conditions are probably suboptimal and since dispersal is traumatic and may decrease viability, these replating efficiencies will be underestimates of self renewal. If the damage sustained in dispersion is a function of colony size then this may introduce a systematic error into the results which cannot be quantified.

*Radiation survival curves*

Tumour cells were irradiated at a concentration of $10^6$ $ml^{-1}$ in 5 ml plastic tissue culture tubes. These were placed in a plastic test tube rack and surrounded by ice and water in a plastic container. The tubes in their container were irradiated using a parallel opposed pair of fields on an isocentric cobalt 60 Theratron unit at a dose rate of $\sim$1.1 Gy $min^{-1}$. Samples treated to varying doses were plated in triplicate at $5 \times 10^5$ cells per dish in liquid or semi-solid conditions as described above. Larger numbers cannot be used because it is not possible to make out colonies when the background of single cells becomes too dense.

### Results

*A comparison of colony formation in semi-solid and liquid medium*

Table I illustrates the clonogenicity of 25 tumours in the two different culture conditions: Agar/Agar and liquid medium/Agar. Plating efficiencies in liquid medium were generally rather higher than

**Table I** Clonogenicity of 25 tumours under the two different culture conditions

| | | *Plating efficiency (Colonies $10^{-6}$ viable tumour cells)* | |
|---|---|---|---|
| *Tumour* | *Patient* | *Agar/Agar* | *Liquid medium/Agar* |
| Malignant melanoma | 1 | 52±2 | 220±26 |
| | 2 | 0 | 0 |
| | 3 | 40±4 | 360±40 |
| | 4 | 136±12 | 370±60 |
| | 5 | 40±12 | 98±4 |
| | 6 | 0 | 0 |
| | 7 | 0 | 0 |
| | 8 | 50±6 | 140±4 |
| Transitional cell carcinoma of bladder | 9 | 596±18 | 940±40 |
| | 10 | 0 | 0 |
| | 11 | 348±36 | 636±84 |
| | 12 | 36±8 | 66±12 |
| | 13 | 852±28 | 970±48 |
| | 14 | 58±12 | 254±40 |
| Carcinoma of breast | 15 | 12±6 | 12±6 |
| | 16 | 668±76 | 364±68 |
| | 17 | 0 | 624±32 |
| | 18 | 430±40 | 636±40 |
| Carcinoma of colon | 19 | 46±8 | 40±4 |
| | 20 | 0 | 0 |
| | 21 | 0 | 0 |
| | 22 | 98±8 | 560±42 |
| Squamous cell carcinoma of cervix | 23 | 0 | 0 |
| Sarcoma | 24 | 0 | 0 |
| Carcinoma of endometrium | 25 | 36±4 | 182±8 |

Results are mean of triplicate plates. ± s.d.

those in semi-solid medium. There is a strong correlation between clonogenicity in the two systems ($r_s = 0.90$). In only one case did we observe growth in liquid medium when growth did not occur in semi-solid medium and in no case did the converse occur. Overall, 64% of our tumours produced countable colonies.

*Linearity studies*

Figure 1 illustrates the relationship between the number of cells plated and colonies formed in liquid medium in a case of well differentiated transitional cell carcinoma. Within a limited range the relationship between cells plated and colonies formed is linear. We have also obtained similar results in one case of breast cancer and 2 melanomas for which data are not presented.

**Figure 1** The relationship between cells plated and colonies formed for a well differentiated transitional cell carcinoma of the bladder, (●; Agar/Agar, ○; liquid medium/Agar). Points are means of colony formation in 3 plates ± one standard deviation. A colony is defined here as a unit of 40 cells or more.

*Radiation survival*

Figure 2a illustrates the radiation survival of moderately differentiated transitional cell carcinoma of bladder in liquid medium over an agar underlayer. The curve is essentially a negative exponential with a small initial shoulder. The Do was ~1.6 Gy and the extrapolation number was 3 consistent with the origin of the colony from a maximum of 3 cells. Figure 2b shows the radiation survival curve of the same tumour assayed in the Agar/Agar system. The Do was ~2.2 Gy with an extrapolation number of 3. The errors are very large and only a limited number of dose points could be studied because the

**Figure 2** (a) Radiation survival of a moderately differentiated transitional cell carcinoma grown in liquid medium/Agar system. Points are means of colony formation in 3 plates ± one s.d. A colony is defined here as a unit of 40 cells or more. (b) Radiation survival of the same tumor assayed in the Agar/Agar system.

number of cells available for study is limited in any individual case. The data are presented only to demonstrate that, qualitatively, the radiation survival curve obtained in the liquid/Agar system resembles that of many other mammalian cells and this suggests that we are indeed counting colonies rather than passively formed aggregates of cells. The apparent difference between the slopes of the survival curves is well within the range of experimental error and other cases have not yet been studied. Unfortunately it is not possible to extend the survival curve beyond 2 logs because the plating efficiency is low and the number of cells which can be plated cannot be increased further due to the difficulty of observing colony growth against a dense background of single cells.

*Colony size and PE2*

Figure 3 shows the size distribution of proliferative units from 2 cases of moderately differentiated transitional cell carcinoma of the human bladder at 21 days after plating. Beyond this time we have observed no further growth in this system (data not presented). In each case small clusters of cells predominate. In case B there is a discrete peak formed by units in which at least 5 generations of cell division have occurred. This is not observed in case A. Figure 4 illustrates the probability of a primary unit containing at least one cell capable of forming a secondary colony in the single colony dispersion and replating experiments carried out in the same 2 cases. The probability of secondary

**Figure 3** The frequency distribution of proliferative units of different sizes derived from 2 moderately differentiated transitional cell carcinomas. Unit size is given in terms of cell number counted by eye up to 32 cells and calculated from 2 colony diameters above that size.

**Figure 4** The relationship between secondary colony formation and primary unit size for 2 moderately differentiated transitional cell carcinomas of bladder. (Primary unit size distributions for the same 2 cases were shown in **Figure 3**). Unit size is given in terms of cell number counted by eye up to 32 cells and calculated from 2 colony diameters above that size.

colony formation increases with the size of the primary colony in each case.

Figure 5 shows the relationship between size of primary proliferative unit and number of secondary colonies formed for case B. The larger the primary unit, the more secondary colonies are likely to be formed on replating. Small clusters, however, do contain cells capable of secondary colony formation and, since small clusters are common, these contribute significantly to overall replating efficiency.

Figure 6 illustrates the relationship between secondary colony size and the size of the parent colony for case A above. The Spearman rank correlation coefficient, $r_r = 0.25$ ($P = 0.05$) suggesting that a small but significant part of the variance in secondary colony size is attributable to variation in primary colony size.

| Number of secondary colonies | 5–8 | 9–16 | 17–32 | 33–64 | 65–128 | 129–256 | Total |
|---|---|---|---|---|---|---|---|
| 5 | 0 | 0 | 0 | 0 | 0 | 1 | 1 |
| 4 | 1 | 1 | 2 | 3 | 1 | 1 | 9 |
| 3 | 0 | 0 | 3 | 4 | 3 | 0 | 10 |
| 2 | 0 | 2 | 2 | 7 | 5 | 0 | 16 |
| 1 | 7 | 6 | 8 | 5 | 0 | 1 | 27 |
| 0 | 35 | 14 | 15 | 19 | 9 | 3 | 95 |
| Total | 43 | 23 | 30 | 38 | 18 | 6 | 158 |

Size of primary proliferative unit

**Figure 5** The relationship between primary unit size and number of secondary units formed for a moderately differentiated transitional cell carcinoma of bladder. Unit size is given in terms of cell number counted by eye up to 32 cells and calculated from 2 colony diameters above that size.

**Figure 6** The relationship between primary unit size and the size of secondary units formed when primary units are disaggregated and replated.

### Discussion

Primary clonogenicity, using a 40 cell colony size cut off point, identifies any cells which are capable of dividing 5 or 6 times in culture. In tumours which arise from tissues where a larger number of generations separate the stem cell from the terminally differentiated cell this assay will identify differentiating transit cells with high levels of residual proliferative potential as well as true stem cells (Mackillop *et al.*, 1983). One way of looking for greater proliferative potential is to isolate the cells formed in the primary colonies and replate them, to find out if they are capable of further proliferation. The system described here is based on the primary culture of tumour cells in liquid medium with an agar underlayer and we have shown that, despite theoretical objections to this type of procedure, the data are similar to those obtained in the traditional semi-solid cultures. We have shown a strong correlation between growth in liquid medium and growth in semi-solid conditions which suggests that clumps are not frequently confused with colonies in liquid culture. Strict adherence to colony size criteria may be important in this context. The first order kinetics of radiation survival in liquid culture are consistent with a single cell origin of the colonies observed although growth from small clusters cannot be ruled out. The linear relationship between cells plated and colonies formed in liquid medium makes the method suitable for use as a quantitative assay but the mobility of the colonies makes counting more difficult than in semi-solid conditions and in this respect the semi-solid system is clearly superior.

It has been previously predicted that, if human tumours contain differentiating "transit" cells with finite proliferative potential in addition to stem cells, it may be possible to distinguish between these two cell classes by analysing the size distribution of colonies formed in culture. While the size of colony formed by a stem cell is limited only by culture conditions, the size of colony formed by a transit cell is inherently limited to an extent dependent on its position in the cell renewal hierarchy (Mackillop *et al.*, 1983). Our deterministic and rather simplistic model suggested that the colony size distribution might be biphasic with the stem cell colony peak separate from the large numbers of small colonies and clusters formed by the transit cells. One of the two bladder tumours studied here produced such a distribution, and, using a simple single colony transfer system, we have been able to study the replating efficiency of primary units of varying size. It has been shown that, although the larger primary units are more likely to contain cells capable of further proliferation, small clusters which have stopped growing may also contain cells with sufficient proliferative potential to form secondary colonies on replating. It is therefore not possible to use colony size distributions as a measure of the ultimate proliferative potential of human tumour cells. The cause of growth arrest in the small colonies which still have proliferative potential is unknown. Inherent variation in rate of cell division may influence the final colony size as may varying ability to grow in suboptimal conditions as the medium becomes exhausted. Only 25% of the variance in secondary colony size is accounted for by variation in size of the primary unit which suggests that heritable variations in growth properties are not a sufficient explanation for the size distributions observed.

Supported by grants from the National Cancer Institute of Canada (W.J.M.) and the Medical Research Council of Canada. J.P.B. is a Research Fellow of the Quebec Cancer Research Society.

### References

BUICK, R.N., MINDEN, M.D. & McCULLOCH, E.A. (1979). Self-renewal in culture of proliferative blast progenitor cells in acute myeloblastic leukemia. *Blood*, **54**, 95.

BUICK, R.N. & MACKILLOP, W.J. (1981). Measurement of self-renewal in culture of clonogenic cells from human ovarian carcinoma. *Br. J. Cancer*, **44**, 349.

BUICK, R.N. & FRY, S.E. (1980). A comparison of human tumor cell clonogenicity in methycellulose and agar culture. *Br. J. Cancer*, **42**, 933.

HAMBURGER, A.W. & SALMON, S.E. (1977). Primary bioassay of human tumor stem cells. *Science*, **197**, 461.

LABOISSE, C.L., AUGERON, C. & POTET, F. (1981). Growth and differentiation of human gastro-intestinal adenocarcinoma stem cells in soft agarose. *Cancer Res.*, **41**, 310.

MACKILLOP, W.J. & BUICK, R.N. (1981). Cellular heterogeneity in human ovarian carcinoma studies by density gradient fictionation. *Stem Cells*, **1**, 355.

MACKILLOP, W.J., STEWART, S.S. & BUICK, R.N. (1982). Density/volume analysis in the study of cellular heterogeneity in human ovarian carcinoma. *Br. J. Cancer*, **45**, 812.

MACKILLOP, W.J., CIAMPI, A., TILL, J.E. & BUICK, R.N. (1983). A stem cell model of human tumor growth. *J. Natl Cancer Inst.*, **70**, 9.

MEYSKENS, F.L., SOEHMLEN, B.J., SAXE, D.F., CASEY, W.J. & SALMON, S.E. (1981). *In vitro* clonal assay for human metastatic malignant melanoma. *Stem Cells*, **1**, 61.

PIERCE, G.B., SHIKES, R. & FINE, L.M. (1978). Tumors as caricatures of tissue renewal. In *Cancer: A Problem of Developmental Biology*. Prentice-Hall: New Jersey, p. 27.

SALMON, S.E. (1980). *Cloning of Human Tumor Stem Cells*. Alan T. Liss: New York.

SELBY, P., BUICK, R.N. & TANNOCK, I. (1983). A critical appraisal of the human tumor stem cell assay. *N. Engl. J. Med.*, **308**, 129.

STANNERS, C.P., ELICIERI, G.L. & GREEN, M. (1971). Synthesis of ribosomal RNA in mouse-hamster hybrids. *Nature* (New Biol.), **230**, 52.

STEEL, G.G. (1977). *Growth Kinetics of Tumors*, p. 217. Clarendon Press: Oxford.